# Professional Male Soccer Players’ Perspectives of the Nutrition Culture Within an English Premier League Football Club: A Qualitative Exploration Using Bourdieu’s Concepts of Habitus, Capital and Field

**DOI:** 10.1007/s40279-024-02134-w

**Published:** 2024-11-03

**Authors:** Wee Lun Foo, Emma Tester, Graeme L. Close, Colum J. Cronin, James P. Morton

**Affiliations:** 1 Research Institute for Sport and Exercise Sciences (RISES), Liverpool John Moores University, Byrom Street, Liverpool, L3 3AF UK; 2 Tottenham Hotspur Football Club, Enfield, London, EN2 9AP UK

## Abstract

**Background and Aim:**

Professional soccer players’ self-reported dietary intakes often do not meet recommended sport nutrition guidelines. Although behaviour change models have previously explored barriers and enablers to nutritional adherence, the cultural factors influencing players’ nutritional habits also warrant investigation. Accordingly, we aimed to explore players’ perceptions of the nutrition culture within the professional soccer environment.

**Methods:**

An interpretivist paradigm, which emphasises that reality is subjectively and socially constructed, underpins this study. Qualitative, face-to-face semi-structured interviews (comprising open-ended questions) were conducted with purposively sampled male soccer players from the English Premier League (EPL) (five British, five migrant; mean age: 26 ± 6 years; mean EPL appearances: 106 ± 129). Data were abductively analysed using thematic analysis according to Bourdieu’s concepts of habitus, capital, field and doxa practices.

**Results:**

This study revealed five key themes: (1) players’ habitus, as shaped by familial, ethnic and religious backgrounds, influences their dietary habits; (2) social capital, via managers (head coaches), teammates and online influences, impact players’ dietary practices; (3) the increase in both soccer clubs’ and players’ economic capitals has advanced nutrition provision; (4) an unequal distribution of economic capitals has led to hierarchical practice in the performance nutrition field with personalised nutrition being somewhat enacted at the higher levels; and (5) body composition measurement is a ‘doxic’ practice in professional soccer that warrants challenge.

**Conclusions:**

Soccer players’ habitual nutritional practices are influenced by personal upbringing and the club context, including economic resources and social capital from managers. The performance nutrition field within professional soccer is also shaped by stakeholders’ doxic beliefs surrounding the perceived optimal body composition of players, with managers exerting social capital.

## Key Points

**Table:** 

Professional soccer players’ dietary practices are profoundly shaped by habitus, stemming from their familial, ethnic and religious backgrounds, and this habitus plays a crucial role in determining success in the nutrition sub-field, as those whose habitus does not align with professional soccer norms often face significant struggles.
Players and clubs with high economic capital can leverage their financial resources to enhance their cultural capital, leading to better nutrition provision, which further amplifies the disparity between high and low economic capitals, as seen between clubs in higher and lower divisions.
In the nutrition sub-field within professional soccer, the doxic belief in ideal body composition standards dictated by the coaches leads to biased players evaluations, favouring those who conform while marginalising others and exacerbates inequality as players with high social capital receive leniency while those with lower social capital face stricter scrutiny.

## Introduction

In 2020, the Union of European Football Association (UEFA) published an expert consensus statement outlining the latest evidence-based nutritional guidelines for elite soccer. Such guidelines suggest that daily carbohydrate (CHO) intake should vary between 3 and 8 g kg^−1^body mass (BM) day^−1^, adjusted according to the energetic demands and specific objectives of the upcoming training sessions and associated fixture schedule [1]. Players are also advised to consume approximately 1.6 g kg^−1^BM day^−1^ of protein (distributed evenly across four servings of 0.4 g kg^−1^BM throughout the day), while dietary fat intake is recommended to fall within the range of 20–35% of total energy intake [1]. Although dietary assessments from elite soccer players (albeit using self-report methods) typically demonstrate that players consume sufficient dietary protein and fat, daily CHO intake often falls short of the recommended guidelines [2, 3]. For example, in evaluating a cohort of players from the English Premier League (EPL), self-reported CHO intake on the day before and in the acute recovery from match play did not align with the goal of optimising muscle glycogen availability and re-synthesis, respectively [4].

The specific factors influencing players’ apparent lack of adherence to nutritional guidelines are not well understood, especially when considering that determinants of food choices in athletic populations are multi-faceted and highly complex. Indeed, such factors are likely to encompass considerations such as individual athlete performance and health objectives, the stage of the competitive season, athlete experience, cultural background, sex, nature of the sport, and the associated food environment [5]. When evaluated through the lens of behaviour change models (e.g. the Capability, Opportunity, Motivation and Behaviour, COM-B model) [6], researchers have explored the barriers and enablers to adhering to nutritional guidelines in professional soccer environments [7, 8]. Indeed, in female soccer players, personal beliefs regarding the impact of CHO on body composition and body image (i.e. reflective motivation), external pressures from social media and coaches’ influences on body composition assessments (i.e. social opportunity) were all identified as factors contributing to a culture of under-fuelling [7]. In male academy soccer players, insufficient food provision in both training and home environments (i.e. physical opportunity) as well as limited education provision from sports nutritionists (i.e. psychological capability) were also identified as potential barriers to nutritional adherence [8].

Prior to evaluating the nutritional behaviours of elite players, it is also of interest to initially explore the embedded nutrition culture within the specific environment, recognising that culture in itself has the potential to shape behaviours [5, 9]. In this regard, Bourdieu’s connected concepts of habitus, field, capital and doxa practices provide a theoretical lens to collectively evaluate how culture influences an individual’s dietary habits [10]. In that sense, habitus refers to the internal disposition such as predispositions to certain eating habits, stemming from each individual’s upbringing and cultural backgrounds [11]. These dispositions may have been influenced by individuals with different forms of capitals; for instance, those with high economic capital (e.g. money and assets) are more likely to have access to higher quality foods, promoting healthier dietary habits. Other capitals, encompassing social (e.g. networks and relationships) and cultural (e.g. knowledge and skills) capitals [12], may also exert considerable sway over an individual’s dietary behaviours. For example, soccer players with greater cultural capital, such as nutrition knowledge, are shown to have better dietary intake [13], which equips them to meet the demands in the field of professional soccer more effectively. The field of professional soccer represents a social arena with its own unique logic and commonly accepted structure [14], where coaches, support staff, nutritionists and other stakeholders compete for various forms of capitals. Resources are unevenly distributed within the field, creating hierarchies not only between clubs and countries, but also within clubs across departments, meaning that players do not have equal access to these resources. Over the past decade, the nutrition sub-field has undergone significant transformation, driven by the ongoing power struggles among key stakeholders. As sport science has become standard for gaining a competitive advantage, managers who were once sceptical of performance nutrition [9] now acknowledge its importance for players’ performance and health [15]. Historically, doctors were the primary sources of nutritional advice due to the limited access to nutritionists [9]. However, today, performance nutritionists are fully integrated in club multi-disciplinary teams [16], a shift supported by their formal accreditation, which has legitimised their role over non-qualified individuals, solidifying their authority in the nutrition sub-field. Within a field, doxa refers to the unquestioned, taken-for-granted beliefs and assumptions that reinforce and legitimise the established dietary norms [17]. These norms are upheld and perpetuated by the cultural beliefs and experiences of influential figures with significant capitals, such as coaches, rather than being grounded in evidence-based practices [18]. Nevertheless, the increasing integration of nutritionists within the soccer field provides an opportunity to challenge these traditional practices. Given this shift, it is crucial to critically examine and address these entrenched doxic practices, steering the nutrition sub-field towards more effective and evidence-based nutritional strategies that improve player health and performance.

To date, only one study has used Bourdieu’s concepts to explore the intricate interplay between culture and the dietary practices of soccer players [9]. In using a cohort of male professional players from the English leagues, this research surfaced the conflicts between players’ ingrained eating habits, as shaped by their family upbringing, versus the dietary protocols advocated within their respective soccer clubs. As such, habitus was identified as a major factor impacting a player’s ability to transition to the ‘soccer diet’. Nonetheless, the remaining components of Bourdieu's concepts such as capitals, field and doxa practices were not readily explored. A thorough exploration of these components would offer deeper insights into the various influences on players’ dietary habits. By understanding how each type of capital affects players’ engagement with nutrition differently and examining the ingrained beliefs within the nutrition sub-field, we can better understand the impacts on players’ dietary practices. It is also noteworthy that this study was conducted during the 2006/07 and 2007/08 seasons and that the nutrition culture and nature of service provision have changed considerably since then. Indeed, the nature of the performance nutrition landscape has likely evolved due to the EPLs increasingly diverse composition [19], substantial growth in financial resources [20] and the rise of sport science and medicine within the professional game.

With this in mind, the aim of the present study was to qualitatively explore professional soccer players’ perspectives of the nutrition culture within the EPL. This study sought to provide an overview of the contested nature of the nutrition sub-field in professional male soccer, identify key figures shaping nutrition culture in the EPL and examine doxic practices prevalent within the field. To this end, we utilised an interpretivist paradigm to underpin our approach (which emphasises that reality is subjective and socially constructed) where semi-structured interviews (comprising open-ended questions) were conducted with purposively sampled adult male players from the EPL. Data were abductively analysed using thematic analysis according to Bourdieu’s concepts of habitus, capital, field and doxa practices. It is hoped that the present data may subsequently inform education and training programmes that strive to improve the quality of execution of performance nutrition services within professional soccer.

## Methods

### Research Philosophy and Positionality

The philosophy of this study was underpinned by an interpretivist paradigm, which emphasises that reality is subjective and socially constructed [21]. This paradigm operates under a relativist ontology, suggesting a belief in the existence of multiple realities for multiple actors within the studied situation. It asserts that these realities can be investigated and interpreted through human interactions involving both the researcher and the participants. This approach emphasises the understanding of the individual and their interpretation of the world around them. To address our aims, we undertook a qualitative investigation aimed at comprehending the experiences and perspectives of individuals within complex social environments [22]. The sampling, data collection and data analysis procedures outlined below were designed to offer a trustworthy and transparent portrayal of the nutrition culture in a single club in the EPL. This study adhered to the Standard for Reporting Qualitative Research (SRQR) recommendations [23].

### Participants

To gain a comprehensive understanding of the perspectives surrounding nutrition culture in professional soccer, male first-team players from a single EPL club were purposefully invited to participate in this study. This methodology mirrors previous qualitative inquiries into nutrition practices within the professional soccer domain [7, 8]. This is typical of qualitative studies where the aim is not to generalise from a large sample, but rather to gain in-depth understanding from a small sample purposefully selected due to their experiences. Participants were approached in person to recruit them for the study, with the details of the study explained during these discussions. A total of ten male EPL players (five British, five migrant; mean age: 26 ± 6 years; mean EPL appearances: 106 ± 129, mean time spent at the current club = 7 ± 5 years) were interviewed. Migrant players are defined as professional soccer players who move from their home country to another country to pursue their careers. Following the principles of qualitative research, the sample size was not pre-determined but instead determined by the data analysis process, with recruitment ceasing after information richness has been achieved by obtaining diverse and in-depth perspectives on nutrition culture in professional soccer from players with varied cultural background [22, 24]. The study was approved by the ethical committee of Liverpool John Moores University, and as condition of this, further details of the participants are not provided to avoid direct identification. All participants provided verbal and written informed consent before completing the interview.

### Procedures

All ten participants engaged in semi-structured interviews (mean 44 min; range 29–74 min), with an ‘open-ended’ approach [25], where questions were posed in a conversational and informal manner to encourage voluntary input and detailed responses [26]. The questions (see Table 1) were informed by the study aims, and Bourdieu’s concepts of habitus, capitals, field and doxa practices. For instance, initial questions were neutrally framed such as ‘What are your thoughts on…?’. Subsequently, probing questions were used to elicit further insights [27]. This format of enquiry enabled participants the freedom to express their experiences and opinions and to guide the discussion towards areas they deemed significant [28]. Moreover, the life history interview method [29] offered a longitudinal view of participants’ careers, delving into transitions, pivotal moments and ongoing experiences. Consequently, the study’s findings extend beyond the scope of participants’ current club, providing a comprehensive understanding of their involvement with various clubs and national teams. To assess the suitability of the interview questions, pilot interviews were conducted with two professional players from the same club. On the basis of the feedback from the senior co-authors for these pilot interviews, the wording of some questions was revised. Pilot interviews were not included in the analysis. All interviews took place in a private office at the club’s training facility and were audio-recorded, then transcribed verbatim. The interviewer was well versed in the professional soccer sub-culture, having worked as a performance nutritionist in the industry for the past 4 years. While this familiarity could potentially bias the interviewer’s approach, it was considered advantageous due to their fluency in understanding the players’ jargon and informal language, and their ability to develop rapport with participants [30]. To prevent leading questions, strategies such as piloting interview questions and utilising open-ended questions were implemented.

**Table 1:** Players’ interview guide and aims

Interview questions	Prompts	Aims
Domain 1: participant background and current dietary practice		
Q1: Can you tell me about your journey as a soccer player so far? Q2: How are training and games going for you now? Q3: What does a typical day of eating look like for you? Q4: How do your eating habits change on days when you have a game compared to training and rest days?	F1: Clubs, age started, setbacks, injuries F2: Any challenges F3: Main meals, snacks, supplements F4: The day before and the day after macronutrient content, types of food, hydration	A1: To understand their background and experience A2: To understand their current perceptions about their performance during training and games A3: To understand their current dietary practices A4: To understand the impact of competition on their dietary practices
Domain 2: life course changes in dietary habits of professional soccer players		
Q1: Can you tell me more about your eating habits during the time when you began playing soccer in your youth [Use information from Q1 in Domain 1]? Q2: How have your dietary habits evolved throughout your playing careers? Q3: What has impacted these changes?	F1: Key influences, provision of foods, main sources of dietary advice F2: Transition into first team, transfers, food provision F3: Why do you think that?	A1: To understand their dietary practice in their youth soccer career A2: To explore the changes in their dietary practice throughout their careers A3: To understand what have influences the changes in their dietary practice
Domain 3: influence of players’ capitals on their food choices		
Q1: Could you explain what influences your current food choices? Q2: Have you experienced any nutrition challenges? Q3: Is there any support you have received to overcome these challenges that has been helpful?	F1: What has impacted these? F2: When? Why? F3: Why? What else would be helpful?	A1: To understand their current determinants of food choices A2: To understand what nutrition challenges they face and why A3: To understand what they perceive to be useful

### Data Analysis

The principal investigator transcribed all interviews. An abductive approach was taken to explore the data. This analytic procedure entailed a series of inducive and deductive processes, acknowledging the interpretative creativity when applying a theoretical framework to participants’ experiences [22]. A six-stage process of thematic analysis was utilised [31]: (1) familiarisation and immersion of the data was achieved through repeated reading and listening during the transcription process; (2) a systematic initial coding process was conducted to identify relevant content; (3) initial codes were reassessed to detect data patterns and generate preliminary themes related to the theoretical framework; (4) identified themes were reviewed for their appropriateness by comparing them with the raw data; (5) upon reaching consensus on the themes, they were refined, defined and named; and finally, (6) data excerpts from each theme were selected to present a concise, coherent, logical, non-repetitive and engaging narrative that reflects the data’s story, both within and across themes. The final author, unacquainted with the club and not involved in the interview process, acted as a ‘critical friend’, who independently checked and challenged data analysis, theme generation and presentation of selected quotes [32]. The role of the critical friend is not to seek agreement or consensus but to foster reflexivity by questioning each other’s construction of knowledge [33]. For example, the concept of doxa was not initially included in the original framework or identified by the principal investigator, but it emerged organically from participants’ responses and was subsequently uncovered by the critical friend. Nonetheless, it was recognised that the lead author’s involvement within the club and personal interest in the topic introduced a level of subjectivity. However, due to the use of a critical friend who ‘checked and challenged’, this insider knowledge was considered advantageous for understanding the topic within its social context [34].

### Methodological Trustworthiness and Rigour

Several measures, consistent with qualitative methods and interpretivist paradigms [35], were implemented to ensure rigour. These measures included recruiting a diverse sample, using a robust theoretical framework and piloting the interview questions. Additionally, independent members of the research team, separate from the primary author, provided critical feedback on the interview techniques and data analysis process. Through these steps, the team sought to offer credible and transparent insights into the nutrition culture within professional soccer settings. The data analysis process demonstrated high level of rigour, characterised by open discussions among all authors, who acted as critical friends and maintained a reflective approach throughout [35]. The worthiness of this research topic was justified by addressing the gap in evidence and practice within this population [4, 36]. Furthermore, the subsequent results and discussion sections outline five themes along with pertinent quotations from the data, enabling readers to interpret the findings independently and contemplate the applicability to their own circumstances [32]. To improve the credibility of the manuscript, member check was conducted by providing participants with a one-page summary of our interpretations and findings for their feedback [37].

## Results and Discussion

Via a reflexive thematic analysis, five themes were established that illuminate the nutrition culture in professional soccer. Aligned with previous qualitative investigation in elite soccer [38], the themes were elucidated through a discussion section, allowing for the relevant exploration of each theme.

### Players’ Habitus, Shaped by Familial, Ethnic and Religious backgrounds, Influence Dietary Habits of Professional Soccer Players

Habitus is defined as an embodied arrangement of social structures that predisposes an individual to certain actions [10]. In other words, habitus is the lens through which people perceive the world and shapes how they act within it. Habitus is embodied in individuals’ food preferences and tastes [10]. The habitus within the professional players’ dietary habits in this study are primarily formed via the socialisation processes in their families as highlighted by one of the British players:

**[Participant 6]** *My family. My mum is a good cook. She always provides good food. We always tended to eat well and have nutritious meals. It comes from her really. I was very lucky growing up because she’s able to cook really well with different varieties of foods.*

Similarly, family also played a significant role in forming a migrant player’s food preference:

**[Participant 1]** *I have very good habits because my grandmom is a great cook. I was lucky because I ate so well during my childhood. I spent more time in my grandparents’ flat than in my home because my parents were very busy with work. So, I was eating at my grandparents’ house every day. So, I used to recover with the food when I eat at my grandmom’s foods. It is still the same when I went to [new country]. I ate so well. Everything is about olive oil. It was not greasy at all.*

Furthermore, the food preferences of players were also reported to be influenced by their ethnicity and religious backgrounds. Participant 2 acknowledged that ‘my mum is half Pakistani so I had a lot of curries and Asian influences. That has been throughout my childhood. I guess when you get older you realised that how much influence your parents have’. Similarly, participant 9 mentioned ‘I’m from Ivory Coast, West Africa so we have a lot of dishes like placani, almost like planted yam, like a dough mashed things with a little bit of sauce. Then, you have sauce graine, like jollof rice but a bit spicier, like that type of dish. Another one is called sauce grand, rice, chicken and sauce but not as spicy, a bit more orange and with tomato. Then, you have attieke’.

Due to their religious dietary restrictions, players might experience difficulties when moving to a different country:

**[Participant 7]** *As you know, I don’t eat cheese and meat together and I don’t eat pork and seafood like calamari and shrimp. So, sometimes when I moved, I needed to ask every time especially the first time I left home, I was shy and afraid to ask so I’m afraid to eat… Yeah, when you go to the restaurant especially with your teammates, most of the things I cannot eat. For normal stuff, I eat everything but sometimes some dishes I cannot eat so sometimes it’s difficult.*

The inherent inertia within players’ habitus [39] may result in resistance to altering established nutritional practices, even when the surrounding environment shifts. For instance, participant 1 recounted his struggle to adapt to the changes required to increase his protein intake at breakfast: ‘I never changed my breakfast. I tried many times, but it is difficult for me to eat salty foods in the morning. I go for toast with a little bit of butter and jam. On the side, I will have fruits, a cup of tea and a cup of coffee depends on my mood. We tried to find protein somewhere which we can get from a protein shake. I tried omelette but it didn’t go down very well. I tried yogurt as well but again if I eat yogurt very early—I don’t feel well’.

Habitus tends to be relatively stable, it is, however, not fixed. Players can still acquire new dispositions and dietary habits through experiences and exposure to different social environments [14]. For instance, participants who came from a country with minimal nutritional support experienced significant improvements in their dietary habits when moving into a new country:

**[Participant 4]** *It (learning) was more like stuff I should eat and how much the portion sizes are. I started to eat more vegetables. Before I came, I never ate vegetables, I don’t really like to eat them. So, when I came, I started to eat more.*

**[Participant 8]***(When I moved to Italy) I started to get to know more about foods because in Italy, they love foods. They eat amazing food. I started to learn much more about food. I started to eat more pasta. I started to eat a little bit healthier.*

Consequently, there may be a lag in the adoption of new nutritional practices, particularly when the cultural background and food preferences of players conflict with the recommendations promoted at the club [40]. The lag in aligning existing practices with sports nutrition guidelines can be attributed to the inertia ingrained in their habitus, making it difficult for players to fully adhere to the current recommendations:

**[Participant 4]** *At the start, I would probably have a piece of chicken and a lot of potatoes and no veg. That’s the way I would eat at home. I would eat a lot of carbs and no veg. That’s the way it was for a little while.*

Overall, one’s habitus is the result of upbringing and culture and continues to shape the way an individual adjusts to new conditions throughout life. As players are instilled with the cultural values of their upbringing, they often perceive their own eating habits as correct, normal and superior [9]. As a result of this, players may be hesitant to embrace food cultures that deviate from their preferences, which would lead to struggles when transitioning to a different country. Nevertheless, the habitus of the professional soccer players can evolve, particularly when transitioning into a new social environment [14] as exposure to other cultures can broaden tolerance and aid in an understanding of how other people live [41]. Developing a habitus that aligns with the expectations of professional soccer offers players a competitive advantage, as it allows them to embody the dispositions and behaviours valued within the sport. The formation of this habitus is largely shaped by access to various forms of capital, including social, cultural and economic capitals [12].

### Social Capital, via Managers (Head Coaches), Teammates and Online Influences, Impact Players’ Dietary Practices

Social capital, as defined by Bourdieu [12], encompasses the benefits and assets that individuals gain from their affiliations, relationships and ties within a community. It also embodies a type of symbolic authority within social spheres, wherein those with substantial social capital typically wield influence, garner respect and receive acknowledgement within their communities. In professional soccer, our data suggest that managers and coaches tend to have the most extensive social capital in the club, which could subsequently influence players’ dietary practices. Indeed, managers, due to their status and power, often have the ultimate decision-making authority on whether to recruit nutritionists.

**[Participant 1]** *[Manager 1] was the first to introduce nutritionist… The nutritionist gave directions on what to cook and how to cook. The nutritionist will put different options and decide what is allowed or not to the kitchen. They start to make a difference between professional athlete and employee.*

Critically, it is also important to recognise that managers, rather than nutritionists, held the most capital when making decisions related to foods:

**[Participant 5]** *I had some managers who would insist that you have to wait. You can have lunch and then you have to wait 2.5 hours before you can train. I have a manager who would take away certain foods and be very strict on that front. But every manager has their own way of working. It’s not necessarily right or wrong. They’re in charge and their jobs are on the line. If they say something goes then it goes. That’s the way it is.*

**[Participant 6]** *At Club A, the food is amazing there as well. They’re slightly more lenient than Club B is. Like before the game, they sometimes put dessert on. Club B, especially with the manager we just had, he’s very hot on nutrition. Everything has to be quite plain. When I was at Club A, there were definitely more sugary foods available per se.*

Social capital is subsequently converted into cultural capital when soccer players align with the influential figures within the soccer field, in most instances, the coaches, by adopting the expected behaviours and practices:

**[Participant 1]** *The first two seasons, the focus is only on the pitch, not outside of the pitch. When [Coach 1] arrived, he changed everything, including my mind and my way of thinking about football. I start to develop myself on things outside of soccer such as nutrition, gym, injury prevention when I was 27 years old. It was the perfect timing for me as it keeps me to perform until today. I need that. The pitch is not enough.*

Nevertheless, players frequently are expected to comply with the standards set by the coaches:

**[Participant 5]** *When I first started, on match day, you could turn up to the stadium an hour and half before. That’s all. You don’t have to turn up for pre-match. It’s fine. Then, the next manager comes in, he wants everyone to be at the stadium for pre-match. That was when there is a pre-match buffet, but it was very simple – pasta, rice and chicken, kind of like what we have now*.

Furthermore, players discern these standards through their habitus. Players whose habitus did not align as well may struggle to adhere to the strict dietary restrictions expected by the coaches. The inertia of habitus makes it challenging for players to seamlessly integrate these new requirements into their daily lives, highlighting the tension between personal dietary practices and the demands set by coaches within the professional soccer environment:

**[Participant 2]** *Coach 2, he obviously banned butter and other stuff, which is very extreme. But again, just because you do that is not going to improve. It is stupid. Now you don’t have ketchup, it is fine now and now you have a perfect diet. No, there is a place for everything. After a game, you can have something sweet or desserts because you have worked maximally. For instance, after a double session, I want to have an ice cream, of course I can have it as I can did two sessions in the gym. If you have a whole tub, then it is different. I feel food is a huge part of life, I don’t want to restrict myself or sacrifice happiness. You can have foods that taste good and you enjoy eating that is also healthy.*

Teammates appear as another social capital that heavily influence the dietary behaviours of soccer players. Players tend to seek advice from players who have achieved high standards or those who possessed great physique:

**[Participant 8]** *I played in [Club C] with [Player A], he is one of the legends in soccer. He knows almost everybody in the whole world about food because he studied for 20 years. I learnt so much from him, I learnt things about very small details. After that, I started to gain more interest and I have learnt a lot. I have been changing a little bit.*

Gaining insights from an experienced player has the potential to bring about positive changes in dietary behaviour. For instance, participant 9 described:

*She was [Player B’s] ex-chef. Just before [Player B] went to [Club D] in Italy on loan, she was looking for work and I was looking for chef. So, [Nutritionist A] knows [Private Chef A] so she put me in touch with [Private Chef A] and then I spoke to [Player B] if she’s good and he said she’s great… Before [Private Chef A], I worked with two others, but they weren’t great. When I first started with [Private Chef A], she was amazing.*

Nonetheless, players could also be exposed to nutrition advice from senior players that may not be optimal for soccer performance, such as gluten free diet and low CHO diet. Participant 8 shared that ‘after I got injured, I stopped eating pasta and stop eating bread so like no gluten almost. I stopped eating candy. I almost never eat candy now. When I was younger, I did it every day. So, I just took away a lot of sugar. I stopped eating gluten’. Furthermore, participant 3 described ‘he was in great shape so straight away you are like “It must be working for him.” He was bang on with everything he eats from Monday to Saturday. Then Sunday, he will [be] like “I will just eat whatever I want. I will be back super healthy during the week.” He was doing that, but he was super lean. He was very much like low carbs. That’s probably the first time I have seen that’.

It is not uncommon for professional players to seek nutrition advice from the Internet:

**[Participant 2]** *It was teenage me Googling or going to YouTube. It is the basic understanding of the myths or what you need to do. Like low-fat, fat-free stuff is maybe not necessary the right thing to do. You can’t do things super quick; everything takes time. You can eat well.*

Players may be at risk of receiving misguided nutrition guidance propagated by unqualified online influencers, potentially leading to adverse effects on their health and overall performance.

**[Participant 8]** *I followed [Influencer A] on Instagram. He’s the one that talks really good about raw meat. I haven’t tried it honestly because people say it can damage you. But I see soccer players eat it, so you never know. So far, I have never tried it.*

In summary, Bourdieu’s concept of social capital reveals the influential role of social status on soccer players’ dietary practice within the professional soccer community. Managers and coaches, possessing considerable social capital, wield influence over decisions related to soccer players’ dietary practices. The players then convert this social capital into cultural capital by adhering to the standards and behaviours expected by the coaches. This strategic alignment signals to key gatekeepers, such as coaches, that the players meet the established standards, thereby demonstrating professionalism and respect within the field. This aligns with previous research identifying coaches as dominant figures endorsing cultural beliefs on athletes’ diets [18], with athletes often regarding them as trusted sources of nutrition information [42]. Furthermore, this study highlighted that players whose habitus does not closely align with the field’s expected norms may struggle to meet these standards. Such challenges can impede their ability to convert social capital into cultural capital, placing them at a disadvantage within the hierarchy of the soccer field. Consequently, this dynamic leads to an uneven playing field, where certain players are more favourably positioned to succeed, thus reinforcing existing social stratifications within professional soccer. Additionally, teammates play a pivotal role in shaping players’ dietary habits, as seen where healthy eating behaviours among teammates influenced others to follow suit [42, 43], due to wanting to comply with what is socially acceptable [44–46]. Influences may also extend beyond the team environment, as some players may turn to social media for nutrition advice, possibly exposing themselves to potential misinformation from unqualified sources. The potential of social media to compromise health and performance is also highlighted amongst other team sports athletes [47]. While social capital can facilitate positive changes, the diverse sources of influence underscore the need for critical education and informed decision-making among soccer players.

### The Increase in Both Soccer Clubs’ and Players’ Economic Capitals Has Advanced Nutrition Provision

Economic capital, which includes money, resources and assets [12], stands out as a significant factor influencing the nutritional habits of professional players. At the individual level, economic capital allowed the players to build their own support team around them. This is not surprising given that EPL players earned an average of £3.1 million per year [48]. The heightened economic capital facilitates the conversion into cultural capital, enabling the players to access personal chefs or meal preparation services. This advantage enhances their ability to optimise nutrition, thereby providing them with a performance edge over players with less economic capital:

**[Participant 9]** *A lot of players here have chefs. A lot of players here have physio as well and so do I. As you are getting older, in your career, time is running out as well. So, you want to maximise everything in a short career. It’s not uncommon that a lot of people here will have a chef.*

**[Participant 4]** *For me, it was just easy because I didn’t have to cook. I didn’t have to make snacks. I have everything in the fridge that I need. If I come back from training and I go hungry, I have some snacks that they will send me. I can just take out the fridge and eat. For dinner, I can just put in a microwave and eat it. For me, it’s just easy. I know it’s good food as well.*

Furthermore, there has been a remarkable increase in revenue within clubs. The top 20 soccer clubs generated more than €10.5 billion in the 2022/23 season [20]. The increase in economic capital has led to a significant improvement in nutrition provision in soccer clubs. Previously, the clubs were not able to afford foods pre- and post-training as described by participant 3: ‘There is no breakfast or lunch. So, you had to eat at home’. Now, EPL players are served with a wide range of high-quality foods:

**[Participant 3]** *Compulsory breakfast…just order when you turn up. Like get me one omelette, poached eggs so it will be that. Training will be at 10.30 am, done by 12.30 pm on the pitch. And then, just go up for lunch when you want to go up. There was literally like a buffet, to be fair, really good quality food. Each day they will do it like a live station, they will make different meals each day.*

**[Participant 3]** *The quality of the food got a lot better. The big thing there was probably post-game. When I first went there, there was pizza and that kind of stuff whereas by the end, there was lasagne. For the home game, there will be hot foods to be cooked fresh after. There was probably the big change to go away from after the game you can just eat whatever you want like pizza, wedges, tomato ketchup to actually eating a nutritious meal.*

Certain clubs have adopted a menu-based system, enabling players to select their own foods. Additionally, players are given the options to take home any meal of their preference:

**[Participant 10]** *It’s exactly like a restaurant. People came up to you with the food, you said what you wanted. Imagine if today you say no chicken, but fish and they will make fish for you. If you want rice or pasta, they will make it for you. There’s always something you can eat and want to eat so I really like it. Also, I can say that I want to eat this tonight and they will make it for you, and you can take it back home. For me, it was perfect.*

In essence, economic capital significantly influences professional players’ dietary habits by affording them access to amenities such as personal chefs or meal services. Access to higher quality food provision enables players to better meet the physical demands and expectations of professional soccer, helping them to secure and maintain top position within the field. In contrast, players with less economic capital may face significant disadvantages, struggling to compete on an uneven playing field. The considerable earnings of EPL clubs reflect the increased economic capital in the sport, leading to better nutrition provision. This shift has expanded food choices, introducing menu-based systems where players can choose meals and take them home. Such improvements highlight economic capital’s role in enhancing players’ performance and overall health, as financial burden is often cited as a major challenge to optimal dietary practice [49].

### An Unequal Distribution of Economic Capitals Has Led to Hierarchical Practice in the Performance Nutrition Field, with Personalised Nutrition Being Somewhat Enacted at the Higher Levels

Field is a social arena that is organised around specific types of capital [50]. Data from this study revealed that the field of performance nutrition has undergone swift evolution due to the changes in economic capitals mentioned in the preceding theme. However, there exists a hierarchy in nutritional provision resulting from the unequal distribution of economic capital. For instance, our data show that there is a clear disparity between the level of nutrition support between academy and first-team players. First-team players have access to high-quality foods with more variety and periodised options tailored to their individual needs compared with academy players, who have a one-size-fits-all, standardised provision:

**[Participant 6]** *When I come over to the first-team site, there’s a lot more options. The academy side has less options. It’s a massive upgrade when you come up to the first-team site. You’re able to pick from a much wider variety of things so you don’t end up getting bored or eating the same thing. In the academy, I think sometimes they don’t appreciate the portion sizes in terms of you might have a game, but it was not very specific. Then, when you get into the first team, it’s more specific about certain days, the portion sizes will be bigger, you know how it works. More carbs and less carbs depending on where you are in the week.*

**[Participant 5]** *When I was in the academy, we didn’t have breakfast, we only had a lunch meal post-training and it’s kind of dependent on where we were training on that day. We didn’t have a training ground. It’s just the first team that has the training ground. It happened for most of my time in the academy. A little pasta box from a local café was our lunch.*

Moreover, in other soccer leagues with less economic capital relative to the EPL, sports nutrition support frequently receives minimal attention or recognition. For example, participant 2 reported that when he was on loan in Sweden, ‘there was no nutritionist and no food at the training ground. I make everything myself, breakfast, lunch, and dinner’. Furthermore, in Germany, there was a limited variety of food options available for them to choose from, as described by participant 10:

*You don’t have a lot of choices to get your food. Not like here, where you can say I want this, and I want this to take home. There were 5–7 things that you could grab. If you want to take food home, you have to take from these seven things, they are not making anything new for you.*

Economic capital between and within clubs therefore plays a significant role in shaping the development of performance nutrition in soccer clubs. It highlights a hierarchical discrepancy in nutrition provision between academy and first-team players, with the latter receiving superior and personalised support. These disparities also extend to different soccer leagues, particularly those outside of the EPL, often providing minimal nutritional support. These findings correspond with a recent audit, which highlighted that category one soccer academies in the United Kingdom, benefitting from greater economic capital, are able to employ more full-time nutritionists, provide more service hours and offer better on-site nutrition compared with lower-category academies [51]. The ability to invest in superior nutrition services allows wealthier clubs to convert economic capital into cultural capital, thereby gaining a performance advantage. This competitive advantage not only reinforces their dominant status within the soccer leagues, but also contributes to increased revenue generation [20]. Consequently, a hierarchy emerges between higher and lower league clubs, with wealthier clubs advancing while lower league clubs struggle, leading to stagnation and a widening performance gap. Additionally, similar hierarchical differences in economic capital are evident between elite male and female soccer players, with female players experiencing subpar food quality and unmet dietary needs [7] in contrast to their male counterparts. These findings emphasise the importance of economic resources in influencing performance nutrition and underscore the necessity for fairer distribution of nutrition resources across the sport.

### Body Composition Measurement Is a ‘Doxic’ Practice in Professional Soccer that Warrants Challenge

According to Bourdieu [11], doxa is a form of social knowledge that is deeply ingrained in individuals and serves to maintain and reproduce existing social hierarchies and structures. It manifests in practices that are rarely explicitly articulated or questioned by individuals within a society. Rather, it functions at a subconscious level, shaping people’s perceptions, judgements and behaviours without their explicit awareness [17]. Within professional soccer cultures studied herein, there exists a doxic belief about the ideal body composition for the players. This belief is often driven by the coaches, as suggested by participant 2: ‘It came from the coaching staff. It was very old school. You just had to be low [in body fat]. It is just a broad thing that lower is better’. Furthermore, doxa can lead to biases in the evaluation of players on the basis of their body composition. In this culture, players who do not meet expected body composition standards may be seen as unprofessional or lazy, whereas players who fit the perceived ideal body type may be favoured over those who do not. This can perpetuate stereotypes and limit opportunities for players who fall outside of the normative body standards:

**[Participant 2]** *But I think high body fat is often perceived as you are eating bad, or you are lazy.*

**[Participant 5]** *There is a thing in soccer where people’s body fat is analysed and they use that as be-all and end-all of how fit or how professional or how ready you are for the game. In a high-stress and high-performance environment, something small like body composition being a little bit too high, for some managers, it can change their mind on a player and change their opinion on how the players live their lives.*

Subsequently, managers could exert significant influence over the food provision on the basis of their beliefs about body composition by dictating the options available to players at training ground and introducing new body composition measurement methods:

**[Participant 6]** *He really cares about body fat. The DEXA has been introduced and the food has completely changed. We have to start ordering from the app. Before, it was always the buffet and we selected what we wanted. He was the most on it. To be fair, my body fat came down so much. We became really fit but I do think that was perhaps a little bit overboard. He really wants to control everything.*

Furthermore, managers will enforce a certain body composition target that reflects their doxic belief:

**[Participant 2]** *When I was younger, I was told that my body fat needed to be below 10%. They used to be very strict about it. Of course, naturally people are different, we have someone in the team who ate terribly but their body fat is super low. If it was me, I could go up and down a lot. It doesn’t take much to change it.*

The pressure to attain unrealistic body composition standards is a manifestation of the symbolic violence exerted by the coaches. This pressure induces stress and anxiety among players, as exemplified by participant 5: ‘I know that some players, not necessarily myself, if they are in a position where their body composition are a bit high, they get stressed about it and their anxiety is high because they think that they could influence the manager’s decision if they play game’. These entrenched doxic beliefs within the field necessitate conformity, compelling players to modify their dietary practices, which are shaped by their habitus, to align with the desirable body composition standards.

**[Participant 2]** *It was difficult when we were in digs. When we left here, we were living in host accommodation. I got home and I had nothing to do. I was hungry. When I got nothing to do, I was bored, I just wanted to eat. I tried to not have snacks.*

Furthermore, players with low embodied cultural capital such as those who are not naturally lean face clear disadvantage in meeting these body composition standards. This lack of embodied cultural capital can place additional pressure on them, as they must put in more efforts to meet the same physical expectations as those who have benefitted from more favourable conditions.

**[Participant 3]** *I had this last year as well when I first came in, they said “Oh, we’ll get you under xx kg.” But I have not been under xx kg since I was 19. I actually felt so much worse about it. You literally do no carbs to go down to that target. I didn’t feel good about it. I didn’t feel strong. I didn’t feel like I had energy to train.*

Conversely, individuals with cultural capital, such as those raised in a supportive family environment, often develop a positive habitus that aligns more easily with the expectations in professional soccer. As participant 6 described, ‘it was never a massive moment where I was like “Wow, I need to change completely”. I always eat relatively healthy… There was never a big moment that I have to change. I was fortunate that my mum was always able to cook really well and always make sure I’m eating healthy, eating fruit and vegetables’. This upbringing, embedded with optimal nutritional practices, enabled participant 6 to effortlessly meet the body composition standards expected in soccer, as he stated, ‘my body fat and skinfold have always been quite low’.

In our experience, while body composition is presented as an objective measure of a player’s physical readiness, it can sometimes be applied selectively to justify or overlook certain players’ performances. For instance, players with significant social capital within the club, such as star players or team captains, may receive more leniency, where their body composition results are either downplayed or disregarded entirely. In contrast, players lacking this social capital might face stricter scrutiny, with their body composition being used as a decisive factor in evaluating their performance or commitment. This selective application of standards can mask favouritism or entrenched biases, as the focus of body composition, in reality, reflects subjective dynamics between players and coaches. These underlying biases in body composition standards were verified during the process of member checking.

Deeply ingrained beliefs about body composition in professional soccer, shaped by coaches, may have an adverse impact on players’ wellbeing. These beliefs influence food provision and body composition measurement, and can result in public criticism and penalties for non-compliance, causing stress and anxiety. This pressure often induces unhealthy dietary practices and deters players from seeking support from nutritionists due to fear of embarrassment. These observations align with previous perspectives of professional athletes from the United Kingdom, indicating that coach reinforcement on body composition often elicited an emotional response, negatively impacting athlete adherence to nutritional guidelines and perpetuating further negative effects [52]. Similarly, the external pressures to meet body composition targets, coupled with stakeholders’ comments, were identified as key factors influencing dietary practice of professional female soccer players [7]. Furthermore, this study highlights how cultural capital, through the consistent access to healthy foods from an early age, can foster the development of a positive habitus, giving players an advantage in meeting body composition standards with minimal difficulty. This finding is consistent with previous research that reported a significant correlation between nutrition knowledge and fat-free soft tissue mass in professional soccer players [53]. The selective use of body composition standards can perpetuate inequality within the team, as decisions are influenced more by the social capital of the players. This veneer of objectivity may allow biases to persist unchallenged under the guise of fairness and performance evaluation.

## Practical Implications and Future Research Directions

Through the lens of Bourdieu’s concepts of habitus, capital and field, the present data reveal interconnected themes regarding professional players’ perspectives on the nutrition culture, holding significant implications for practitioners in the field. Bourdieu’s concepts offer a robust framework for performance nutritionists, helping them comprehend why players choose certain diets, shaped by habitus, field dynamic, capitals (social and economic resources) and doxa (accepted beliefs). For example, understanding players’ habitus enables nutritionists to tailor recommendations to align with their preferences, fostering sustainable changes. Recognising field dynamics identifies key capitals shaping nutrition culture, while analysing doxa within cultural context sheds light on why certain nutritional practices are favoured or stigmatised. Consequently, the findings from this study provided profound insights into the factors that facilitate or hinder nutritional adherence among professional players [7, 8], thus enabling a more nuanced understanding for practitioners. Armed with this understanding, practitioners can adeptly manoeuvre through the field, developing contextually relevant and effective strategies for fostering positive dietary habits. This study also highlights the power dynamics in EPL soccer [54], depicting the field as a battleground where players struggle for team selection, a process dominated by coaches. Coaches hold significant power, defining values and maintaining team hierarchy, particularly through their belief that all players must meet specific body composition standards. Players with greater cultural capital—knowledge and behaviours valued in soccer—are better equipped to meet these expectations, navigating the professional soccer field more successfully. In contrast, those whose habitus does not align with these norms often struggle, facing barriers that limit their recognition and opportunities within the hierarchy. To address these disparities, clubs and practitioners should enhance players’ access to cultural capital by providing comprehensive nutrition education, high-quality food and supportive environments such as host families to nurture positive habitus from an early stage. These measures can help players whose habitus may not initially align with the field’s expectations, creating a more inclusive environment where all players can succeed. Moreover, our data suggest that practitioners entering a new sport setting would benefit from gaining a thorough understanding of the nutritional culture within that context before introducing any nutritional interventions. Furthermore, universities and professional nutrition-accrediting bodies should also provide guidance to prepare aspiring practitioners to comprehend ‘sports nutrition culture’. This may entail integrating Bourdieu’s concepts and/or other theoretical models into university curricula, accrediting body competency frameworks and sport-specific practitioner programs that are endorsed by governing bodies (e.g. the EPL). The present study did not include interviews with key stakeholders to avoid information overload and maintain our primary focus on players’ experiences. Future research should address this gap by examining the perspectives of key stakeholders on nutrition culture in professional soccer, considering the influence individuals can exert on others’ behaviour, as suggested in social conformity theory [55]. Of particular interest is investigating how body composition has become a deeply ingrained doxic practice within elite soccer. Additionally, the purposive sampling method used may be a potential limitation, as our findings might only reflect the culture and procedures at one club and those willing to participate. Future research should therefore aim to develop a better understanding of the prevalence of such findings within other soccer club settings. Nevertheless, this study allows readers to reflect on this study and assess whether the findings resonate with their experiences, the specific setting they operate in and the people with whom they interact [35].

## Conclusions

Our data demonstrated several critical components of the current nutrition culture within professional soccer. Firstly, habitus emerges as a foundational element, deeply rooted in familial, ethnic and religious backgrounds, which significantly influences players’ food preferences and habits. Additionally, social capital, primarily wielded by managers, coaches, teammates and online influences, significantly impacts soccer players’ dietary practices. Moreover, economic capital drives advancements in nutrition provision within clubs, enhancing players’ access to improved nutrition provision. Nonetheless, the hierarchical nature of the performance nutrition field underscores the unequal access to nutritional support, particularly between academy and first-team players and across various leagues. Finally, the pervasive influence of doxic beliefs regarding body composition standards perpetuates biases, leading to public criticism, penalties and stress among players, fostering unhealthy dietary practices and reluctance to seek support from nutritionists. In essence, the findings emphasised the multi-faceted nature of nutrition culture within male professional soccer. Thus, it is imperative for practitioners to grasp the nutrition culture within their own context to facilitate effective implementation of any nutritional intervention. On the basis of this study, Bourdieu’s concepts provide a comprehensive framework for practitioners to understand the complexities of athlete’s dietary choices within the specific sport and environment they are working in.

## Declarations

### Availability of data and material

The manuscript has no associated data.
